# Prediction of Performance in Standardised Assessments from Computer-Based Formative Assessment Data

**DOI:** 10.1007/s10758-025-09947-2

**Published:** 2026-02-23

**Authors:** Benjamín Garzón, Stéphanie Berger, Charles C. Driver, Martin J. Tomasik

**Affiliations:** 1 Institute of Education, University of Zurich, Zurich, Switzerland Kantonsschulstrasse 3, 8001; 2 Institute for Educational Evaluation, Zurich, Switzerland

**Keywords:** Computer-based formative assessments, Summative assessments, Machine learning, Online assessments, Large-scale assessments

## Abstract

Summative assessments (SAs) and formative assessments (FAs) fulfil complementary functions in the educational endeavour. SAs measure knowledge at the end of a unit in a standardised, high-stakes setting, while FAs evaluate student performance during daily classroom activities to tailor feedback and instruction. Computer-based FA (CBFA) systems enable collecting unprecedented amounts of data objectively and with minimal disruption for students, under conditions that more closely resemble real-life behaviour. Given concerns about student stress and ecological validity associated with SAs, potential biases in teacher judgements, and the high burden entailed by traditional classroom assessments, we investigated whether and how well FA outcomes can predict SA outcomes. Specifically, we estimated student abilities in a large sample of children evaluated at different time points during compulsory schooling and performed a systematic comparison of regression models trained to predict SA abilities on different subsets of features derived from FA abilities and auxiliary variables. A model that included mean abilities in different competence domains performed best, accounting for a considerable proportion of variance (30–48%), although this was still below that explained by past SA measures. Most predictive FA features generally corresponded to abilities from the same or a similar competence domain as the predicted SA ability. We report systematic model biases that would warrant consideration when using the models for decision-making. Our findings provide valuable insights into how learning progress connects to future achievement, which can help teachers adapt instruction earlier and inform policies to reduce reliance on high-stakes testing.

## Introduction

The aphorism “if you cannot measure it, you cannot improve it”, often attributed to the brilliant mathematician and physicist Lord Kelvin (Houser, 2022), rings particularly true in education. Assessments are a crucial and highly effective part of education (Gibbs & Simpson, 2005), used to measure students’ abilities and progress, provide feedback, inform teachers’ decisions, structure coursework, maintain the quality of educational activities and evaluate the performance of educational programmes and reforms. However, because assessments determine examinees’ future academic and career opportunities, at times with few chances of redress afterward, they are also a source of stress and anxiety (Denscombe, 2000). Long-standing controversies around the need for assessments in the face of their downsides (Hendricks, 1947; Marchand, 2004; Wyness, 2021) have been re-ignited after the Covid-19 pandemic prompted the modification, deferment, or cancellation of standardised exams in many countries, among numerous other disruptions to classroom activities (Huong & Schwabe, 2022). This tension between the need to monitor student performance impartially and the cost and inconvenience that such undertaking entail motivates the present study. Specifically, little is known about how data from low-stakes, ongoing assessments can serve as valid proxies for high-stakes outcomes.

### Formative Assessments and Summative Assessments

Educational assessments are generally divided into two broad paradigms which differ in both operationalisation and purpose, formative assessments (FAs) and summative assessments (SAs; Dixson & Worrell, 2016; Dolin et al., 2018; Harlen, 2005; Harlen & James, 1997). On the one hand, FAs are assessments carried out as part of ordinary classroom activities and intended to help teachers monitor students’ learning with the aim of improving their academic outcomes (Tomasik et al., 2018). Information about students’ progress allows teachers to modify goals timely, adjust daily activities and give feedback (Van der Kleij et al., 2015). Certainly, quality feedback that provides constructive suggestions for improvement has been repeatedly demonstrated to enhance learning (Bangert-Drowns et al., 1991; Black & Wiliam, 1998; Bransford et al., 2000; Glazer, 2014; Yorke, 2003). As they are naturally incorporated in everyday activities, FAs facilitate transparent participation, whereby teachers can acquire a comprehensive picture of learners’ mistakes and weaknesses (Knight, 2002) and gain insights into the difficulties and misunderstandings they encounter (Harlen & James, 1997), ultimately helping to craft educational experiences that promote learning with understanding (Harlen & James, 1997).

To warrant gathering detailed and extensive information about the students, FAs should be administered frequently. This is time-consuming for teachers, who may additionally not be adequately trained to perform rigorous, objective evaluations (Black et al., 2010; Brown, 2013). Fortunately, digital technologies are providing new tools to tackle these challenges. A particularly effective example of the use of digital technologies in school is given by computer-based formative assessment (CBFA) systems, which are software platforms designed to support tasks of data collection and evaluation in the classroom (Moser, 2016). CBFAs provide an opportunity to enhance the psychometric rigour of FAs (Tomasik et al., 2018), and have the powerful advantage over more traditional educational tools of enabling the acquisition of rich longitudinal data sets. These can be leveraged to understand individual progress and tailor teaching strategies to students’ needs.

The main function of SAs, on the other hand, is to gauge how much of the administered material has been acquired by examinees at the end of an educational unit (e.g., a school grade or an entire school period), and this results in a number of important differences with respect to FAs. SAs tend to have a broader scope and are administered in standardised settings, and are high stakes for examinees, meaning that their scores carry (often important) consequences for them (G. F. Madaus, 1988), namely deciding which future educational and/or professional pathways they will gain access to. For this reason, *washback*, or the impact of assessment on teaching and learning (Alderson & Wall, 1993), is more severe in SAs (Abrams et al., 2003; Biggs, 1998; Stobart & Eggen, 2012), as in high-stakes contexts both instructors and learners have strong incentives to focus excessively on the material anticipated for the test (Lazear, 2006). Washback can result in more superficial learning (Baird et al., 2017; G. Madaus & Russell, 2010), which is at odds with the goals of educational practice (Abrams et al., 2003). Students generally harbour negative perceptions on traditional assessment methods that incentivise a narrow, procedural engagement and prefer more innovative methods that support learning with lesser requirements of stress and effort (Iannone & Simpson, 2017; Struyven et al., 2005; Traub & MacRury, 1990).

As everyday situations normally occur in low-pressure conditions, performance in low-stakes assessments may reflect typical performance more closely than in high-stakes assessments, the estimates of which should be more related to the examinees’ maximum capacity. Maximum and typical performance may be only weakly correlated, and motivational as well as personality factors play an important role in the distinction between the two, which is particularly relevant in the workplace (Deadrick & Gardner, 2008; Napolitano et al., 2021; Sackett et al., 1988; Turner, 1978). Indeed, test-taking effort is commonly more variable in low-stakes contexts (Barry et al., 2010), which in combination with their lower level of standardisation implies that FA ability estimates are normally less reliable than those derived from SAs. However, this can be partly compensated by the greater density of data afforded by CBFA systems. Besides, effects of environmental factors like pollution and temperature, which have been shown to occur in high-stakes assessments (Ebenstein et al., 2016; Graff Zivin et al., 2020; Park, 2022), are more likely to be diluted when testing is spread over long periods of time.

Given that formative assessments occur naturally within classroom activities and may capture student abilities with less stress and disruption than high-stakes summative assessments, they represent a potentially valuable alternative approach to performance monitoring. In the present work, our aim was to evaluate the effectiveness of performance measures during regular, low-pressure classroom activities in predicting standardised assessment outcomes.

### Predicting Summative Assessment Scores

Through their day-to-day interactions with students, teachers form an opinion of how much progress each student is making, both in relation to themselves and the group, and will certainly develop informal expectations about their students’ scores in future SAs. Hoge et al. (1989) reported a moderate to high agreement between teachers’ predictions of achievement and actual standardised test scores (median *r* = 0*.*69 across 16 studies, corresponding to *R*^2^ = 0*.*48). Furthermore, teacher perceptions can predict performance significantly in SAs even several years later (Alvidrez & Weinstein, 1999). These observations support the validity of teachers’ judgements of academic achievement (Gerber & Semmel, 1984). However, these judgements are mostly subjective and suffer from a number of downsides. Instructors may vary greatly in their ability to appraise students (Coladarci, 1986; Helmke & Schrader, 1987), for example depending on experience (Mulholland & Berliner, 1992). Teachers leading large classrooms may be less reliable in their predictions, as that will typically result in their having fewer individual interactions with their students (Blatchford et al., 2007). Even if different teachers may resort to similar traits when forming expectations about students, they are likely to ascribe a different level of importance to each of these traits (Geven et al., 2021). Moreover, their estimations may be biased by non-academic factors and sometimes irrelevant information, such as ethnicity and socioeconomic status (Alvidrez & Weinstein, 1999; Burgess & Greaves, 2013; Hansen, 2016; Murphy & Wyness, 2020; Tournaki, 2003), and tend to be more accurate for higher-achieving students (Demaray & Elliot, 1998). The expectations that teachers develop about their students’ potential academic achievement tend to act as.

"self-fulfilling prophecies" (the well-known Rosenthal or Pygmalion effect; Brophy & Good, 1974; Rosenthal & Jacobson, 1968) potentially inflating the correlation between predictions of achievement and actual scores. Despite the richness of teacher-student interactions, these shortcomings prevent teacher judgments from serving as reliable standalone assessments, underscoring the need for assessment practices which are unbiased, fair, and consistent across instructors, grades, and institutions.

Against this backdrop, CBFAs offer a convenient source of valuable data about classroom activities that can be readily converted into objective, quantitative estimates of learners’ abilities (Tomasik et al., 2018). The integration of digital technologies in education, such as computerised adaptive testing systems (Meijer & Nering, 1999;

Veldkamp, 2005; Weiss & Kingsbury, 1984), intelligent tutoring systems (Corbett et al., 1997; Graesser et al., 2012) or the aforementioned CBFAs, has generated vast amounts of educational data that can be used to characterise multiple aspects of learning activities. This has spawned entire research fields, including educational data mining and learning analytics (Batool et al., 2023; Romero & Ventura, 2020; Slater et al., 2017). Machine learning techniques have become increasingly common in educational research for analysing these rich data sets (see, e.g., Alamri & Alharbi, 2021; Bowers, 2010; Dillon & Stolk, 2012; Garzón et al., 2025; S. Lee & Chung, 2019; Pardos et al., 2014; J. Wang & Yu, 2025). These techniques include both classical supervised and unsupervised methods, as well as methods developed expressly for educational problems, such as knowledge tracing (Abdelrahman et al., 2023; Corbett & Anderson, 1995; Nakagawa et al., 2019).

In view of the drawbacks of SAs and the better capabilities to administer FAs supported by computer-based solutions, the central aim of the present article was to determine if and how well CBFA-based outcomes can predict SA outcomes. Within modern validity theory, predictive relations between assessments provide evidence relevant to both construct alignment and the consequences of score use. On the one hand, there are theoretical reasons to hypothesise that the former should predict the latter. Cognitive abilities tend to be positively correlated across domains (Gustafsson & Undheim, 1996; Spearman, 1904, 1925, 1927), which is usually thought to reflect the existence of a general ability that influences responses to different items. Mathematics and reading skills are strongly associated (Bailey et al., 2020; Chen & Chalhoub-Deville, 2016; Korpipää et al., 2017), with a substantial proportion of the observed correlation due to genetics (Davis et al., 2014). In SAs in particular, a general ability factor has been found to account for most of the variance in student responses (Pokropek et al., 2022a, 2022b; Saß et al., 2017). Thus, if this ability affects both FA and SA outcomes to some degree, a relationship between the two is expected to arise and this commonality should enable predicting the latter from the former. On the other hand, the greater diversity of settings and items found in FAs, together with the differences between FAs and SAs mentioned in the previous section, suggests that scores from these two assessment approaches should diverge.

Previous research supports the existence of a link between FA and SA performance across various educational stages. Croteau (2011) examined mathematics and reading FA data from students in grades 1 to 4, showing a significant, albeit weak, relationship between FA and SA scores accounting for 11% of the variance. Among primary school mathematics learners, performance in FAs accounted for between 8 and 19% of the variance in SAs in another study (C. Wang et al., 2021). A large-scale study on 23,000 students of grades 6 to 8 used FA data collected over the course of a year from a mathematics intelligent tutoring system to predict SA scores (Zheng et al., 2019). The researchers compared the performance of several machine learning models trained on process and demographic variables, achieving an explained variance in the range of 0.6–0.7. In college students, Zhang and Henderson (2015) found that scores from formative quizzes were significant predictors of SA scores. Yang et al. (2024) used decision trees to predict students at risk of failing a final course assessment based on FA data from a learning management system. The ability to predict outcomes using multiple FA tasks was greater than using just one task.

With the exception of the study by Zheng et al. (2019), the aforementioned studies had moderately-sized samples (< 500 students) and focused on a narrow range of school subjects. Here, we leveraged a large-scale data set with over 100,000 primary and secondary school students, covering several competence domains within mathematics, as well as native and foreign language subjects. This allowed us to examine the importance of different features derived from these competence domains. It is often unclear to what extent classical regression methods use all the available information (due to their stringent model structure), so assessing the potential of machine learning approaches that can exploit potential complex nonlinearity is important. Critically, and in contrast to previous research in this field, we used well-established, optimal psychometric models to obtain the ability estimates that provided the inputs for these models.

The present study addresses the following research questions: Firstly, can FA scores predict SA scores, and if so, to what extent? Secondly, what is the importance of features derived from different competence domains? Thirdly, how do FA-based predictions compare to those derived from previous SA scores? Finally, are there systematic biases in these predictions? To answer this questions, we trained machine learning models on features derived from CBFA data on a large sample of children and adolescents to predict the abilities estimated from SAs administered at different time points during compulsory schooling. To gain insight into the importance of different predictors, we performed a systematic comparison of models trained on different subsets of features and identified those features that were most important to predict different competence domains. Finally, we put our results into perspective by comparing them to predictions based on scores from previous SAs in the same population and discuss the presence of biases when predicting SA outcomes.

## Methods

### Data Set Description

The two online assessment instruments brought to bear in the present work have been used in Northwestern Switzerland over several years to assess students’ ability throughout compulsory schooling.

#### Formative Assessment: MINDSTEPS Data Set

The CBFA system MINDSTEPS (https://www.mindsteps.ch/) was designed to collect objective information about students’ current abilities and learning progress for four school subject domains: mathematics, German (the official language in the region where the schools are located), English and French (the two foreign languages taught). The MINDSTEPS CBFA system serves a population thousands of students in four.

German-speaking cantons of Switzerland and therefore significantly shapes digital learning within and outside the school setting.

The items in the MINDSTEPS item bank cover topics and competences from grade 3^1^ until grade 9, spanning seven years of mandatory schooling. The items of each school subject domain are further categorised in competence domains:’numbers and variables’,’form and space’ and’measures, functions and probability’ for mathematics;’reading comprehension’ and’grammar’ for German;’reading comprehension’,’listening comprehension’ and’grammar’ for French and English, giving a total of eleven competence domains. The CBFA system automatically evaluates students’ responses, with wrong or omitted responses scored as 0, and correct responses scored as 1. The original.

MINDSTEPS data set used here comprised 112,013 students, 40,036 items and 38,301,724 responses, making its size and scope rare if not unique for a data set in this domain. A more comprehensive overview of the data and the development of the CBFA can be found in Tomasik et al. (2018).

The items presented to a student are selected by the teacher or recommended by the.

CBFA system based on the student’s estimated ability. As students from different (adjacent) grades may respond to several common items, those items that have been presented to students from more than one grade can be used to link the different grades and obtain a vertical scale (Hanson & Béguin, 2002). See Berger et al. (2019) for more details about the items and item administration.

#### Summative Assessment: Check-Dein-Wissen Data Set

Check-Dein-Wissen (https://www.check-dein-wissen.ch/) is a standardised assessment providing summative feedback that is administered at four important time points during the students’ school career; i.e., at the beginning of grade 3 and the end of grade 5 and at the end of grades 8 and 9. The four measurement occasions are compulsory for all students in the four cantons where the data were acquired. The assessments are administered each year based on standardised administration guidelines. The assessments are administered on paper in primary school, whereas those administered during secondary school are computer-based. The following competence domains were available for this assessment:’numbers and variables’,’form and space’, and’measures, functions and probability’ for mathematics;’reading comprehension’ and’grammar’ for German;’reading comprehension’ and’listening comprehension’ for French and English (the assessment does not include a’grammar’ test for these languages), giving a total of nine competence domains. The original Check-Dein-Wissen data set comprised 112,742 students, 5,306 items, and 178,256 responses.

**Fig. 1:**
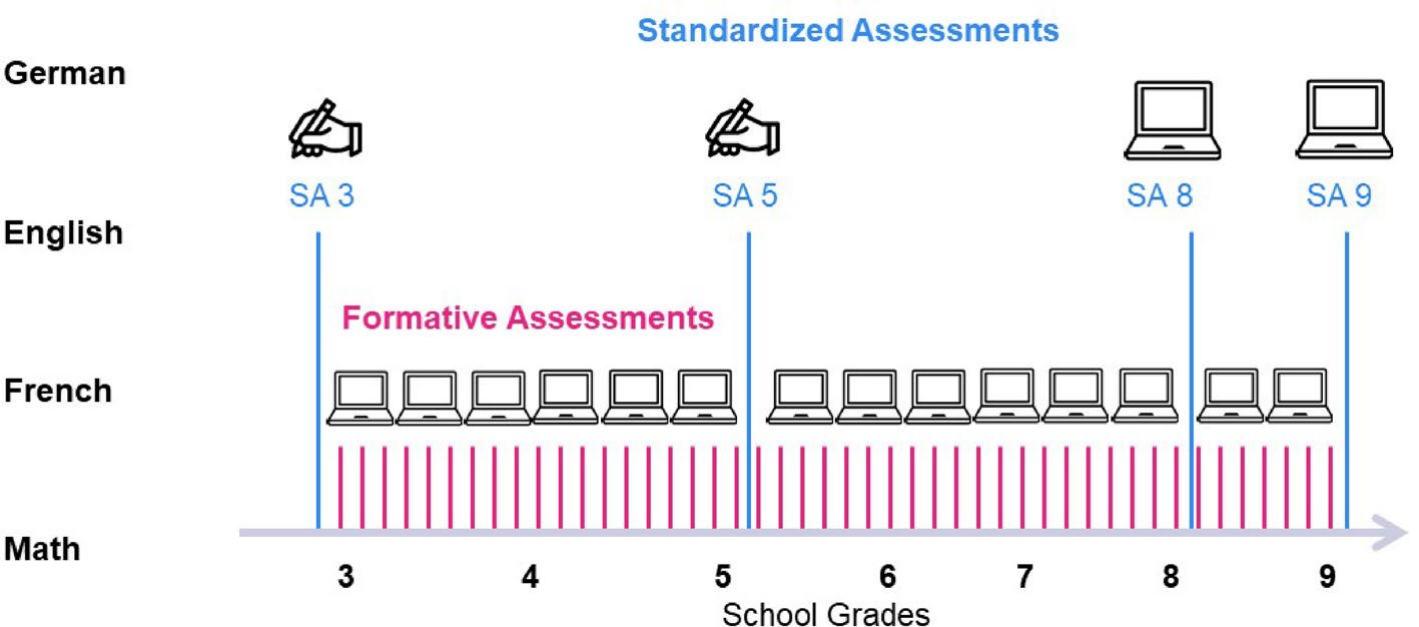
Study design. Summative assessments (Check-Dein-Wissen) were administered in a standardised setting in grades 3, 5, 8 and 9. Formative assessments (Mindsteps) were administered at multiple, irregular, individual-specific time points in variable settings. The assessments covered German, French, English and mathematics. See Table 1 for an explanation of the labels of the different competence domains. Illustration designed with images from Flaticon.com

Figure 1 displays an overview of the assessments over grades and Table 1 the labels used in the article to designate the various competence domains.

**Table 1:** Labels used to designate the different competence domains

Subject domain	Competence domain	Label
German	German reading	dles
German	German grammar	dsif
French	French listening	fhoe
French	French grammar	fsif
English	English listening	ehoe
English	English grammar	esif
Mathematics	Numbers and variables	mzuv
Mathematics	Form and space	mfur
Mathematics	Measures, functions and probability	mgfd

#### Ethics Approval Statement

All procedures performed in studies involving human participants were in accordance with the ethical standards of the institutional research committee and with the 1964 Helsinki Declaration and its later amendments or comparable ethical standards. In line with the American Psychological Association’s Ethical Principles and Code of Conduct, as well as with the Swiss Psychological Society’s Ethical Guidelines, written informed consent from the students and their parents was not required because this study was based on the assessment of normal educational practices and curricula in educational settings. The Institute for Educational Evaluation as a contractor of the cantonal educational authorities committed to obeying the laws of the four cantons involved to ensure strict data confidentiality. In line with the laws of the four cantons, approval from an ethics committee was not required for this study.

### Data Selection and Preparation

CBFA responses were discarded if they corresponded to unfinished assessment sessions. Only students attending public schools and with available age, gender and mother tongue were included in the analyses. In the cases in which a student had repeated the SA (e.g., because they had repeated the grade), only the estimates from the first occasion were included.

To ensure a certain reliability of the model parameters, we removed sessions with less than 10% of correct answers, responses that were particularly fast relative to the group (either faster than the fastest recorded correct answer, or in the fastest 10%), or items with a probability of correct above 95% or below 5% (as these carry little information). Students who had completed less than 50 items were also removed. Although these thresholds are not drawn from prior literature, they were derived from systematic exploratory analyses of our data. In initial sensitivity checks on earlier versions of the data set, we found that the exact threshold did not substantially influence the distribution of ability estimates subsequently derived from the data. These data selection decisions were made prior to any of the regression analyses reported below.

When predicting the outcomes of a particular SA examination, only the FA data of each student from before that examination were entered into the models. Furthermore, we only used student abilities if they had at least three data points available so that we could estimate the mean and standard deviation of the estimates. For the earliest grades, 3 and 5, fewer observations were available, and none had all the necessary features to train all the models (see below for an explanation of the features). Therefore, the analyses we present concern the prediction of SA scores in grades 8 and 9.

### Student Parameter Estimation

We estimated students’ abilities with Item Response Theory (IRT; Embretson & Reise, 2000; Reise et al., 2005), a modelling technique frequently used to score tests (Eggen & Verhelst, 2011). In IRT, the probability of a person responding correctly to a test item is determined by their ability (a latent trait) and the characteristics of the item (item parameters). IRT assumes local independence (correctly answering one item does not affect the probability of correctly answering another) and monotonicity (as a person’s ability increases, so does the probability of a correct response). In the present study, we also adopt the common assumption of unidimensionality, which states that the latent trait varies along a single dimension. IRT encompasses a family of models of varying complexity, determined by the number of item parameters.

We scaled the FA data by fitting a classical two-parameter logistic (2PL) IRT model to the entire data set. The 2PL model was employed in our study because prior analysis of this data set (Tomasik et al., 2022) indicated that using models with additional parameters (3/4PL) did not provide significant improvements in out-of-sample prediction accuracy. According to this model, the probability that the response *u*_*ij*_ of student *i* to item *j* is correct is given by:

1 $$p_{ij}(\eta_j) = p(u_{ij} = 1 | \eta_j) = 1 +\frac{{e}^{{a}_{j}({\theta }_{i}-{d}_{j})} }{1+ {e}^{{a}_{j}({\theta }_{i}-{d}_{j})}}$$

In this expression, *a*_*j*_ and *d*_*j*_ denote, respectively, the difficulty and the discrimination of the item, and *θ*_*i*_ the ability of the student. Parameters for the FA data set were obtained with maximum a posteriori (MAP) estimation as implemented in the R package bigIRT (Driver, 2023), which was expressly developed to handle large-scale data with high sparsity as those that concern the present article. These data were scaled using the same model for the whole data set. SA data were scaled using a separate 2PL model for grades 5 and 8/9. Weighted likelihood estimates were obtained using the R package TAM (Robitzsch et al., 2022). These SA ability estimates were already available from a previous application and were not recomputed, but a comparison of the two methods has shown them to yield very similar estimates when fitting a same data set (Driver, 2023).

### Model Features

We trained regression models to predict SA performance (outcome variable) for the different examination occasions, based on several features obtained from the FA data, or the SA data from previous years (predictor variables). As the data sets were originally gathered for a real-world application, we explored the options available that could be expected to be related to SA performance. To ascertain the added value of considering separate competence domains, we included features that combined ability estimates across domains, as well as features that kept them apart. The features we defined, grouped in sets with common characteristics, were the following:

1. System-use variables comprising the number of previous sessions, years since starting to use the system, frequency of use (i.e., number of previous sessions/years since starting to use the system); and demographic variables: mother tongue (German/non-German) and gender (**g**; 5 features).
2. Last ability estimate across all competence domains, i.e., most recent estimate of any of the abilities estimated (**l**; 1 feature).
3. Last ability estimate separately for each competence domain. This feature represents the most recent estimate of domain-specific abilities (**L**; 9 features).
4. Mean ability estimate across all competence domains (**m**; 1 feature). Here, we averaged all FA ability estimates that were collected before the date of the SA. This provides a more robust feature than the single last estimate, resulting from combining multiple previous estimates.
5. Mean ability estimate separately for each competence domain (**M**; 9 features).
6. Standard deviation of ability estimates across all competence domains (**s**; 1 feature). We included this feature to capture the effect of performance variability.
7. Standard deviation of ability estimates separately for each competence domain. (**S**; 9 features).
8. Intercept and slope of a regression line fitted to ability estimates across all competence domains. This was done separately for each student, with ages mean-centred around the age of SA test-taking so that the intercept approximated the SA ability at that time (**p**; 2 features). By adding these features, we wanted to include simple indicators of ability growth.
9. Intercept and slope of a regression line fitted to ability estimates estimated as in the previous point but separately for each competence domain (**P**; 2 × 9 features).
10. Interactions between all pairs of features included in feature sets 1 and 5 above (**I**; 120 features).
11. Abilities estimated from the last SA examination, i.e., grade 5 abilities as predictors when predicting grade 8 abilities, and grade 8 abilities as predictors when predicting grade 9 abilities (**c**; 9 features). Predictions based on the previous SA examination can be regarded as an underestimate of the reliability of the SA abilities (as students may undergo considerable changes during the interval between consecutive examinations). Still, they constitute a benchmark against which predictions from FA-based models can be compared, as the SA examinations from different time points involve the same competences and are performed under similar conditions.

All models included system-use variables, mother tongue and gender, along with different combinations of the remaining feature sets above. To refer to the multiple models tested in the following sections, we coded them using the bold letters in the list above. For example, a model termed *glP* would have system-use and demographic variables (**g**), last ability estimate across all competence domains (**l**), and intercepts and slopes of regression lines (**P**) fitted separately to estimates of each competence domain; a model termed *glPc* would additionally include the SA ability estimates (**c**) for all competence domains as predictors. Rather than just seeking to find the best model, our goal was to perform a systematic comparison of the different models in order to gain an understanding of the predictive value of the different sets of features. When using features from multiple competence domains, estimates from up to two of them out of the possible nine were allowed to be missing. When modelling ability growth with linear models (list items 9 and 10), we kept only students with at least 10 data points and a minimum age difference between the first and the last estimate of 6 months in order for the estimated intercept and slope to have some reliability. Given the large number of possible combinations of feature sets, in the results section we present only those that are most relevant or interesting. Data preparation and feature generation were carried out with custom Python (python.org).

scripts.

### Regression Models and Evaluation

We trained ridge regression models (Hilt et al., 1977) to predict the SA abilities based on different feature subsets. Ridge regression is a regularised form of multiple regression commonly used in scenarios with multiple correlated predictors. To illustrate that this was the case here, Tables 2 and 3 show the correlations between mean FA ability estimates for grades 8 and 9, respectively. These correlations are moderate and positive for all pairs of competence domains. Ridge regression addresses multicollinearity by adding a penalty that shrinks all coefficients toward zero, which stabilizes the solution when predictors are highly correlated. Data from missing competence domains were imputed based on the remaining features available via multiple imputation. Features were then mean-centred and standardised. The ridge regression model depends on a regularisation hyperparameter *λ* that was tuned with nested cross-validation (10 folds). To evaluate model performance, we calculated the cross-validated coefficient of determination *R*^2^ using 10 folds (i.e., the test data set for each fold comprised 10% of the data).

**Table 2:** Correlations between mean FA ability estimates for grade 8

	dles	dsif	ehoe	eles	esif	fhoe	fles	fsif	mfur	mgfd	mzuv
dles		0.57	0.50	0.52	0.49	0.47	0.50	0.51	0.52	0.53	0.49
dsif			0.47	0.50	0.52	0.43	0.48	0.53	0.51	0.53	0.51
ehoe				0.70	0.66	0.48	0.49	0.48	0.39	0.43	0.41
eles					0.66	0.47	0.53	0.50	0.42	0.44	0.44
esif						0.45	0.51	0.52	0.44	0.43	0.45
fhoe							0.62	0.58	0.40	0.41	0.40
fles								0.62	0.45	0.46	0.43
fsif									0.48	0.46	0.50
mfur										0.64	0.61
mgfd											0.65
mzuv

**Table 3:** Correlations between mean FA ability estimates for grade 9

	dles	dsif	ehoe	eles	esif	fhoe	fles	fsif	mfur	mgfd	mzuv
dles		0.56	0.49	0.52	0.49	0.42	0.47	0.46	0.49	0.51	0.44
dsif			0.44	0.50	0.51	0.40	0.45	0.53	0.48	0.50	0.47
ehoe				0.69	0.65	0.48	0.48	0.45	0.38	0.42	0.35
eles					0.66	0.48	0.55	0.50	0.41	0.44	0.40
esif						0.48	0.50	0.49	0.41	0.43	0.41
fhoe							0.59	0.57	0.38	0.39	0.39
fles								0.60	0.44	0.43	0.44
fsif									0.42	0.43	0.45
mfur										0.63	0.60
mgfd											0.64
mzuv											

For the best-performing model, we repeated the previous procedures with an alternative technique, extreme gradient boosting (XGB; Sheridan et al., 2016), a type of machine learning algorithm that is particularly useful for handling large data sets with many and complex features (similar to the very popular random-forest approach; Breiman, 2001). XGB builds a series of decision trees, with each subsequent tree attempting to correct the errors of the previous one. It is particularly well-suited for structured data with non-linear patterns, delivering state-of-the-art performance. It can handle large-scale, complex data sets more effectively than random forest regression and is more interpretable than alternatives such as deep learning, making it a practical and efficient choice for analysing complex educational data sets.

To reduce overfitting and improve generalisation, XGB employs L1 and L2 regularisation. The hyperparameters that were tuned for this model were the L1 and L2 regularisation parameters, the maximum tree depth and the number of estimators. The remaining steps were identical to the ridge regression models.

We inspected the importance of each of the features entered into the model. Feature importance reflects which features are contributing the most to the predictions of the model. It is calculated for a single decision tree by assessing the amount whereby each feature reduces the model’s loss when the feature is included. The more a feature reduces the loss, the higher its importance. The overall feature importance for the model is the average of the importance values of all its individual decision trees.

Due to data missingness discrepancies across features, the amount of data available to train the models differed depending on which subset of features they were based on. Given that the amount of data used to train a model will impact its performance, to ensure a fairer comparison between models, we trained them in two different settings: first, an imbalanced scenario in which we trained each model with all available data for the corresponding set of features, and a balanced scenario in which we trained all models on the same maximal data set comprised of all students with complete data across all features. All the computations above were performed using custom scripts in Python, version 3.9.13 and R, version 4.2.0.

### Model Biases

After fitting the regression models, we noticed that they tended to shrink their predictions with respect to the observed abilities, such that the slope of the regression line of predicted against observed abilities was smaller than 1 (see Results and Fig. 3). This is a known problem of regression models, commonly referred to as calibration (Van Calster et al., 2019; Van Calster et al., 2020; van de Wiel et al., 2023). When plotting the model predictions against the true values, ideally the data points should lie around the identity line. A model that is not well calibrated produces systematic biases in the predictions, which may become manifest as a non-linear relationship between predicted and true values, or a linear relationship with a slope different from 1, as was the case here. Avoiding such biases is important in real-life applications because they are likely to lead to deficient decision-making. Some multivariate models, such as ridge (Hilt et al., 1977) or lasso regression (Tibshirani, 1996), use regularisation schemes that penalise large coefficients, resulting in shrinkage. Besides, since true abilities follow a bell-shaped distribution, with many more observations close around the mean than far away from it, the model has more data of the former to learn from, resulting in smaller errors for those. We thus tested whether this problem could be alleviated by fitting the model to subsamples of examples for which the distribution of true abilities was closer to uniform. With this aim, we divided the range of true abilities in 100 equally-sized bins and constructed samples by drawing *N*_*B*_ examples from each bin without replacement (or the maximum possible for the bin). For low values of *N*_*B*_, this resulted in a subset of training examples for which abilities had a distribution that was close to uniform (but with less samples than the original data set), and by increasing *N*_*B*_ it was possible to gradually shift towards the original distribution.

Then, as we varied *N*_*B*_, we estimated, respectively, *R*^2^ and the slope *S*_*R*_ of the regression line of predicted against true abilities, respectively, using tenfold cross-validation as above. To compensate for the lower number of samples used to train the model, we repeatedly fitted models to different random subsets thus obtained and averaged the predictions. All these analyses were performed with the subset corresponding to the model *gM* (see section Model Features).

In order to discard that the shrinkage we observed was due to particularities of our data (e.g., the specific distribution of the ability parameters), we created a synthetic data set and repeated the above procedures. In this data set, the true ability *Y* was simulated according to the model:

2 $$Y = X\beta + \sigma_{\epsilon }^{{2}} \epsilon ,$$

with

3 $$X_{i} \sim N\left( {0, \, I} \right),$$

4 $$\beta \sim N\left( {0, \, I} \right),$$

and

5 $$\epsilon \sim N\left( {0,1} \right),$$

where *X*_*i*_ denotes row *i* of *X*, and the size of *X* was equal to the size of the matrix of predictors of the real data set. We simulated true abilities *Y* varying $\sigma _{\epsilon }^{2}$ to examine the effect of having different levels of noise. Finally, so that we could observe the effect of different regularisation strategies, we performed all analyses with three types of regression models: ordinary least squares (OLS; no regularisation), lasso (L1 regularisation), and ridge (L2 regularisation).

## Results

### Comparing Models

**Fig. 2:**
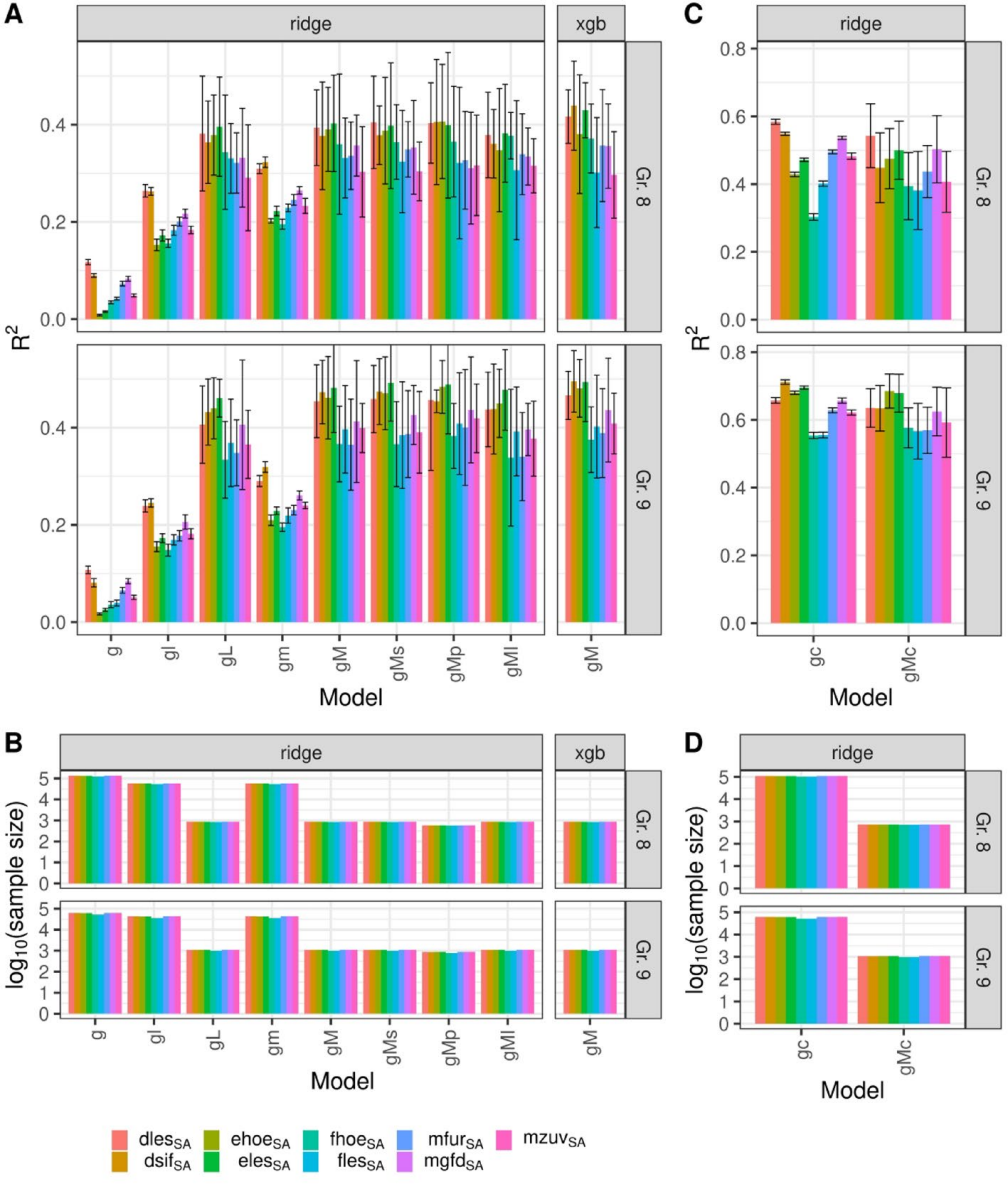
Model performance comparison, imbalanced scenario. **A** Cross-validated R^2^ for each model tested. Error bars denote standard deviations around the mean, showing variation over 10 cross-validation folds. The left facet (ridge) shows the results for the ridge regression models, and the right one (XGB) for the extreme gradient boosting model (only for the gM feature set). The upper row corresponds to grade 8 and the lower row to grade 9 results. **B** Sample size (i.e. number of students, in logarithmic scale) for each of the models shown in (**A**). **C** Cross-validated R^2^ for models including abilities derived from the previous SA, either alone (model gc) or in combination with FA features (model gMc). **D** Sample size for each of the models shown in (**C**). For the analyses shown in this figure we used all available data (i.e., the amount of training observations differs across models, see Fig. 4 for the results in the balanced cases). The features and the coding used to designate the different models are described in the subsection Model Features in the main text. As an example, gM is the model that includes system-use variables and mean abilities separately for each competence domain. The different bar colours indicate the competence domain that was predicted, as shown in the legend below the plots. See Table 1 for an explanation of the labels of the different competence domains. Gr.: grade

Figure 2A shows the cross-validated *R*^2^ for the models tested in the imbalanced scenario. As expected, including ability estimates when training a model clearly improved its *R*^2^ relative to a model based only on information about system-use and demographic variables (*g*). Models based on the mean of FA estimates previous to the SA (*gm/gM*) outperformed those using only the last estimate (*gl/gL*). Using mean estimates for each step separately (*gM*) led to a clear gain in predictive performance with respect to using the global mean across competence domains (*gm*). Adding the standard deviation of the estimates (*gMs*) or the intercept and slopes did not lead to improvements, and therefore henceforth we focus on the *gM* model. This model explained on average between 30 and 40% of the variance in the target variable for grade 8, and between 37 and 48% for grade 9, depending on the domain. Overall, we obtained the best accuracy for the German and English language competence domains and the worst for the French language competence domains. Using a more flexible machine learning technique such as XGB did not lead to important gains in cross-validated *R*^2^.

The number of students that were available for each of the analyses is displayed in Fig. 2B (since we used tenfold cross-validation, 90% of the sample size indicated was used to train a model in each fold; see Methods). Even though there were many more data available for models based on aggregate measures of ability estimates across all competence domains (*gl/gm*; note that the y-axis in Fig. 2B is in logarithmic scale), they were outperformed by models including measures of ability estimates for the different competence domains (*gL*/*gM*).

When training the model on ability estimates from the previous SA (*gc*, Fig. 2C), *R*^2^ reached up to 58% for grade 8 and 71% for grade 9, but there was no meaningful gain when additionally including the FA ability estimates (*gMc*), and the standard deviation of the *R*^2^ values increased markedly. As can be seen in Fig. 2D, there was a considerable imbalance in the amount of data used to train these two models.

**Fig. 3:**
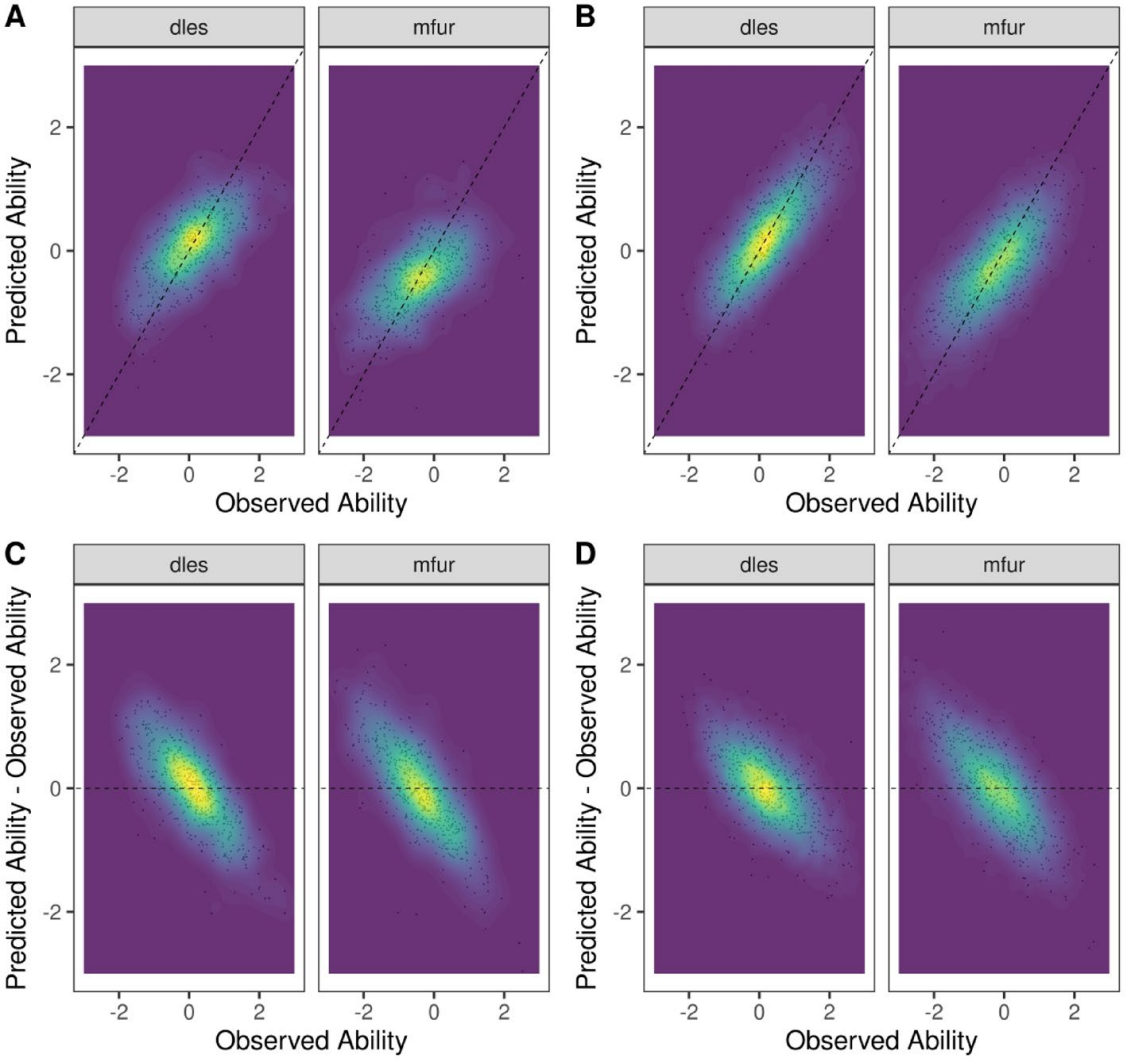
Predicted against observed abilities. For two of the SA outcome competence domains, the plots show the ability predicted by the models gM (**A**), and gc (**B**), respectively, against the observed ability. The difference between the predicted and observed abilities is plotted in **C** (gM model), and **D** (gc model). For visualisation purposes, only a random subsample of 500 data points, drawn uniformly from the different cross-validation folds, are displayed in each scatterplot. Each data point represents one student. The color overlay shows the density of data points, with warmer colors indicating a higher density. The plots corresponding to the competence domains that are not displayed showed very similar patterns. See Table 1 for an explanation of the labels of the different competence domains

Figure 3 shows predicted SA abilities against observed abilities for two competence domains for grade 8, for models (*gM*, panel A) and (*gc*, panel B), respectively. The plots corresponding to the remaining competence domains and grade 9 showed very similar patterns. For all competence domains, both models underestimated the abilities of students with high ability and overestimated the abilities of low-ability students (panels C and D). The predictions of the *gc* model were more accurate and had less bias.

**Fig. 4:**
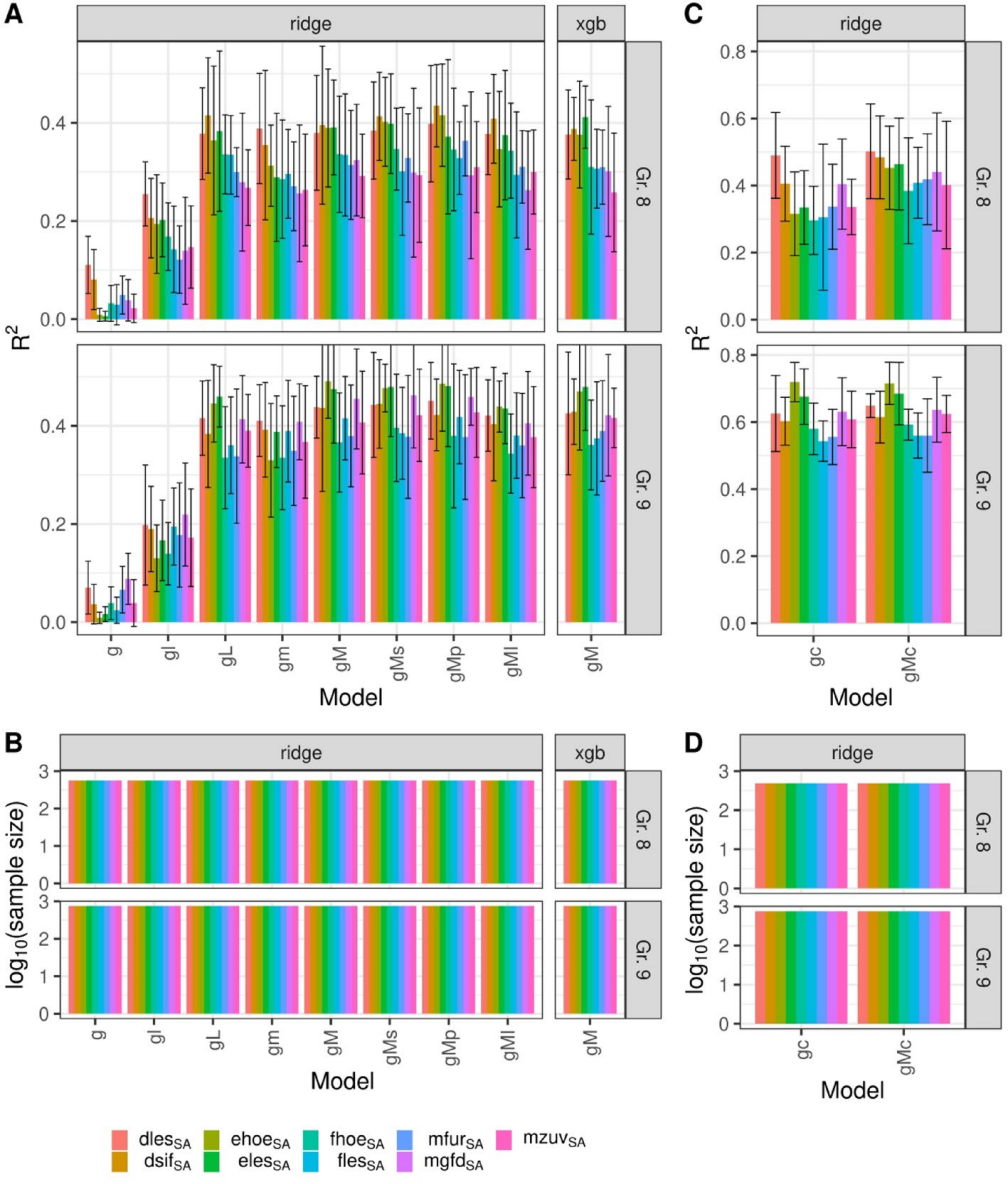
Model performance comparison, balanced scenario. **A** Cross-validated R^2^ for each model tested. Error bars denote standard deviations around the mean, showing variation over 10 cross-validation folds. The left facet (ridge) shows the results for the ridge regression model, and the right one (XGB) for the extreme gradient boosting model (only for gM feature set). The upper row corresponds to grade 8 and the lower row to grade 9 results. **B** Sample size for each of the models shown in (**A**). **C** Cross-validated R^2^ for models including abilities derived from the previous SA, either alone (model gc) or in combination with FA features (model gMc). **D** Sample size for each of the models shown in (**C**). For the analyses shown in this figure we used the same subset of students, for which there were enough features and outcomes available that it was possible to train all the models for a given grade. The features and the coding used to designate the different models are described in the subsection Model Features in the main text. The different bar colours indicate the competence domain that was predicted, as shown in the legend below the plots. See Table 1 for an explanation of the labels of the different competence domains. Gr.: grade

Given the large differences in sample size for the data available to train the different models due to missingness asymmetries across features (Fig. 2B), the models were trained again on a common data set formed by all the observations with the complete set of features (balanced scenario). Cross-validated *R*^2^ scores were generally lower due to the use of a reduced training data set, but adding features to the (*gM*) model again did not lead to clear improvements (Fig. 4A). The worst accuracy was still found when trying to predict the French language competence domains. The advantage of the models trained on ability estimates from the previous SA (*gc*, Fig. 4C) was in this scenario smaller for grade 8 (maximum of *R*^2^ = 49%) but similar for grade 9 (maximum of *R*^2^ = 72%), compared to the imbalanced scenario. Including the FA abilities in the model in addition to the SA abilities (model *gMc*) resulted in improved accuracy for some of the models (*eles*_*SA*_, *ehoe*_*SA*_) for the grade 8 data, but not for the grade 9 data.

### Model Biases

**Fig. 5:**
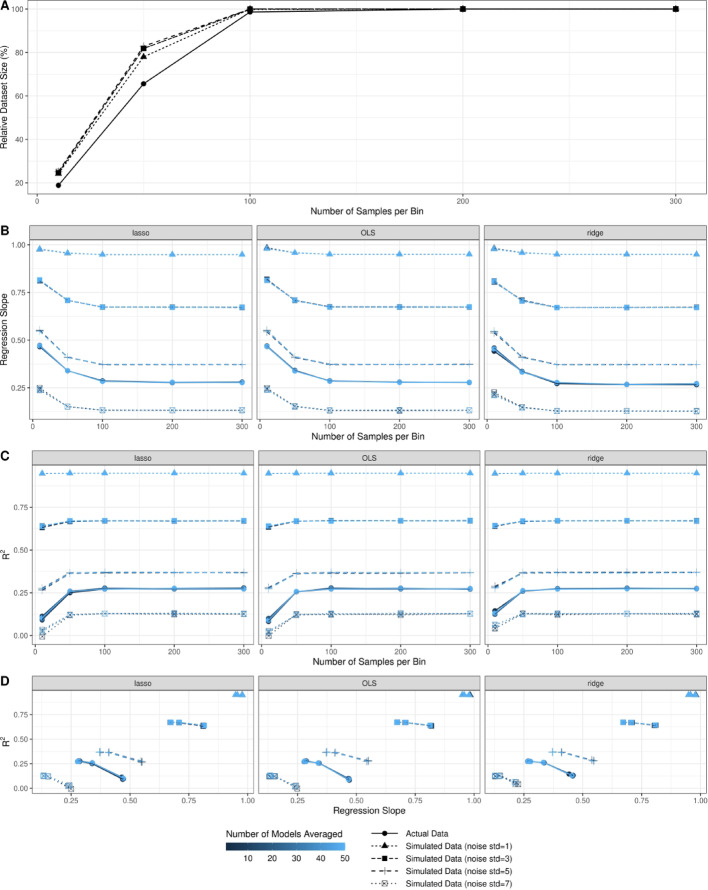
Model performance and calibration. **A** Relative data set size, i.e. percentage of students relative to the original data set, as a function of the number of samples per bin. **B** Dependence of the cross-validated slope of the regression line of predicted against true SA abilities on the distribution of true SA abilities. The fewer samples per bin, the more uniform was the distribution of true SA abilities but fewer samples were available to train the model (see Methods). **C** Dependence of the cross-validated R^2^ on the distribution of SA abilities. **D** There was a trade-off between regression slopes and R^2^, such that better calibration led to worse accuracy. The results were similar for the lasso (left), ordinary least squares (OLS; center), and ridge (right) models

Fitting the regression models to subsets of the training data set such that their distribution was closer to uniform (i.e., increasing the number of samples per bin *N*_*B*_) led to some calibration improvements (Fig. 5A), but at the expense of worsening their accuracy (Fig. 5B) because of the smaller number of data points available to train the model. The regression slopes were still always well below 1, regardless of the number of samples per bin. Only for simulated data with low noise levels were the regression slopes close to the ideal value of 1. Repeating the sampling a number of times and fitting and averaging the ensuing models led only to minimal improvements, and these were only appreciable when the number of bins (and therefore the relative size of the data set used for training) was small.

There was a trade-off between regression slopes and *R*^2^, such that better calibration led to worse accuracy of the predictions (Fig. 5C). Increasing the standard deviation of the noise of the simulated data worsened both calibration and accuracy, shifting the slope-*R*^2^ curve downwards and to the left. The profile corresponding to the real data was similar to that obtained for synthetic data with a large noise standard deviation. The fact that the synthetic data showed comparable patterns for the regression slopes and *R*^2^ (Fig. 5A–C), rules out the possibility that these patterns were merely the result of idiosyncrasies of our data. Lastly, we obtained equivalent results for the lasso, OLS and ridge models; therefore, the lack of calibration was not due to the shrinkage produced by regularisation (as it is not present in OLS).

### Feature Importance

**Fig. 6:**
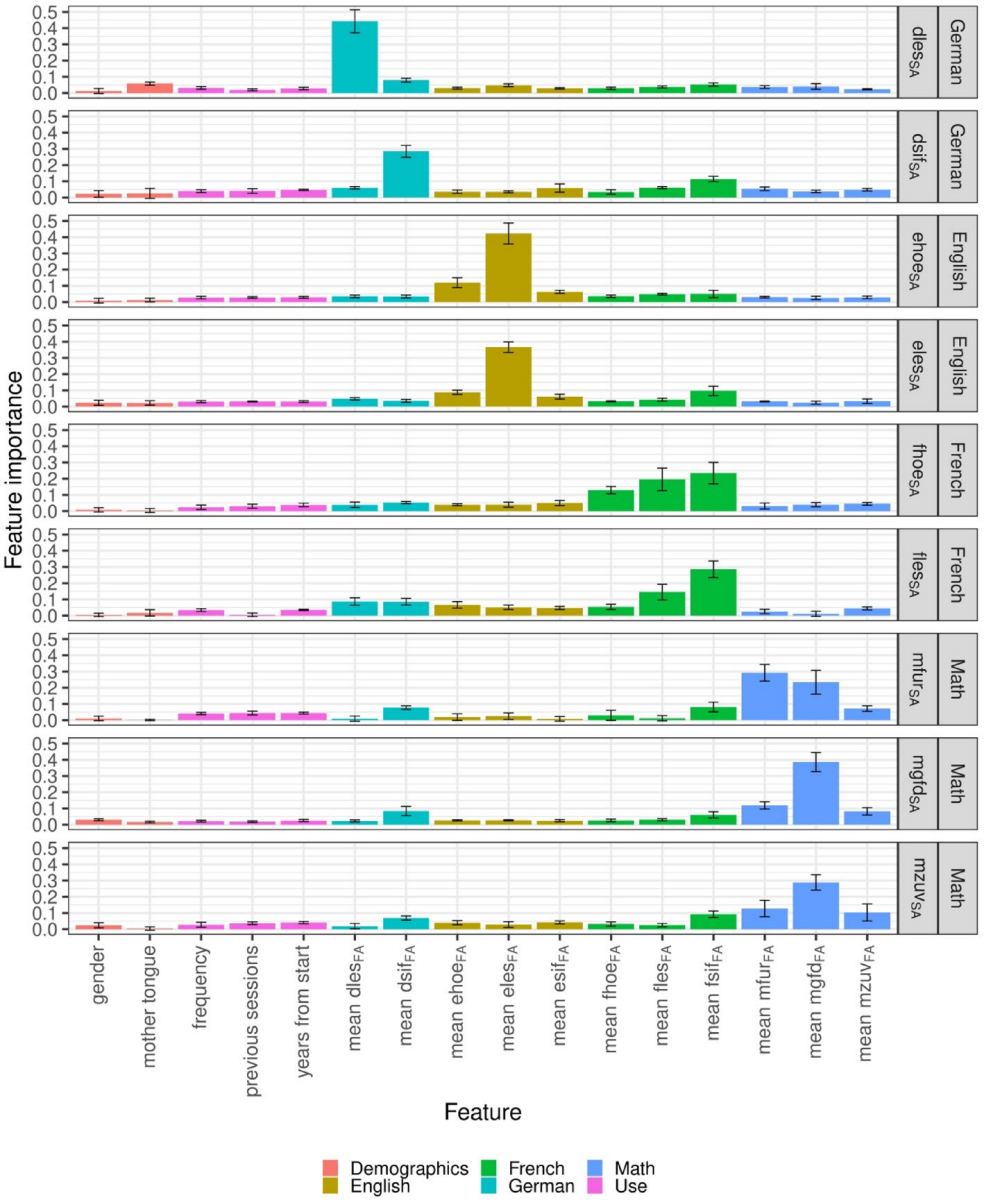
Feature importance. Feature importance for the gM models trained to predict each of the SA competence domain abilities on the full data set. Each of the facets from top to bottom corresponds to one of the SA competence domains that a model was trained to predict, as indicated by the labels on the right side. Features, which include student demographic characteristics, variables relative to the use of the FA system and means of the FA abilities, are grouped according to their type (subject domain in the case of ability features), denoted in different colors. Error bars denote standard deviations around the mean, showing variation over 10 cross-validation folds. See Table 1 for an explanation of the labels of the different competence domains

The importance of the different features in the XGB model can be seen in Fig. 6. Mean FA ability in a certain competence domain had predominantly high importance when predicting SA abilities of the same competence domain. For example, the most important feature in the model predicting SA ability in German reading comprehension (*dles*_*SA*_) was mean FA ability in German reading comprehension (*dles*_*FA*_). Although there were deviations from this pattern (e.g.,’numbers and variables’ [*mzuv*_*FA*_] abilities were not the most important predictor of’numbers and variables’ SA abilities), the predictors with the highest feature importance were always in the same subject domain (in this case’measures, functions and probability’ [*mfgd*_*FA*_]), and the FA abilities in the other competence domains within a given subject domain were also among the important predictors of SA competence domains in the same subject domain (e.g., French reading [*fles*_*FA*_] and grammar [*fsif*_*FA*_] were important predictors of French listening [*fhoe*_*FA*_]). Interestingly, mother tongue was a prominent feature only when predicting German competence domains, emphasising the advantage of native speakers in this domain but not in the others.

## Discussion

SAs and FAs illuminate complementary facets of the educational journey. SAs are administered in standardised settings and provide a snapshot of student status at specific time points during this journey, usually at the cost of being more disruptive of the teaching and learning process. FAs, meanwhile, are less intrusive and provide valuable information about the ongoing learning process and characterisation of performance in ecological conditions. In spite of the discrepancies between these two assessment types, as only a reduced number of cognitive abilities should underlie performance in both scenarios, it is reasonable to believe that the respective outcomes should be associated. Thus, here we investigated if and how well it was possible to predict SA scores from FA scores using a large-scale data set provided by the MINDSTEPS CBFA system. Our results show that predictions based on CBFA abilities accounted for a considerable amount of variance, supporting the (perhaps obvious) notion that common cognitive skills are at play in both assessments. Nonetheless, the proportion of variation explained was still below that predicted by preceding SA measurements (which is bounded above by the reliability of the SA measurements). Interestingly, the FA features showed specificity in the sense that the most predictive ones generally tended to correspond to abilities from the same or a similar competence domain as the predicted SA ability. Even though CBFA systems implement objective data-collection procedures, we observed a systematic bias in the predictions that would need to be taken into consideration when using the models for decision-making purposes.

The predictions of a model based on separate mean abilities for each FA competence domain accounted for up to 48% of the variance in SA abilities. The amount of variance explained was hence of a similar magnitude to that between teachers’ predictions of achievement and standardised test scores (Hoge & Coladarci, 1989). Models that relied on the average of FA predictions performed better than those relying solely on the most recent prediction. This suggests that the reliability gain from incorporating more measurements compensates for the bias towards ability estimates that are more distant in the past, possibly because abilities are relatively stable within the timeframe covered by the FA data set we analysed. The addition of other features such as standard deviations and interactions did not result in sizeable improvements in the amount of variance explained. Perhaps unexpectedly, adding a linear estimate of growth in ability (slopes of the regression lines) did not improve the predictions. Besides being very sparse, the FA data analysed here were noisy due to the larger amount of factors that can influence performance in a non-standardised setting (e.g., location, distractions, time of the day, …). Moreover, since the MINDSTEPS CBFA system is still relatively novel, the age range covered for a given student is short thus far (1.7 years on average), compared to the timescale at which developmental changes occur (the period encompassing childhood and adolescence). Therefore, although the large number of students in the data set can be leveraged to obtain accurate population estimates of ability growth, at the individual level these estimates have a rather low reliability. There was little benefit from including non-linear and interaction effects (explicitly in the ridge model with interactions and implicitly in the XGB model). Although XGB often outperforms linear models, the presence of measurement error may dampen the influence of such effects (Jacobucci & Grimm, 2020). In a similar analysis, Zheng et al. (2019) did not find a marked advantage in using random forest regression over linear regression models. Lastly, the fact that the SA scores are also imperfect estimates of ability influenced by numerous unmeasured factors further hinders our capacity to predict them.

The models based only on SA abilities outperformed those trained on FA data only, as apparent from the analysis where we trained the models on the same subset of the data to make the comparison fair. Adding the FA features to the SA features led only to improvements in accuracy for the grade 8 data, as opposed to the grade 9 data. For the grade 8 prediction, the SA features were measured 3 years before (in grade 5), whereas for the grade 9 prediction they were measured only one year earlier (in grade 8). This difference in the recency of the SA predictors, perhaps paired with an increasing stability of achievement scores with age (K. Lee & Bull, 2016), can explain both the better performance of the grade 9 *gc* model over the grade 8 model, and the fact that including also the FA features led to gains only for the latter. In other words, when the SA acquisition was distant in time, the FA data added complementary information, enhancing the predictive ability of the model.

Comparing the feature importance for models predicting the various SA competence domains revealed an interesting pattern, namely congruity between the most predictive FA subject domains and the predicted SA subject domains, and also largely a good correspondence (albeit not perfect) between FA competence domains and SA competence domains. This demonstrates a substantial specificity of the ability estimates for the different subject domains and (to a lower degree) for the different competence domains. The pattern of feature importances shows also higher correlations between competence domains corresponding to the same subject domain (e.g., the three mathematics competence domains predicted each other). This overall pattern is compatible with a multidimensional structure of the achievement measures formed by abilities with a certain degree of specificity that could be captured by the CBFA measurements and grouped within the subject domains, rather than a single general ability (see e.g., Baumert et al., 2009) for a discussion on this topic), even though commonalities between mathematics and language achievement have been extensively reported (Bailey et al., 2020; Chen & Chalhoub-Deville, 2016; Korpipää et al., 2017) and a single factor may account for most of the variance in achievement (Pokropek et al., 2022a, 2022b; Saß et al., 2017). This presence of specificity in the abilities is further supported by the accuracy gains we observed when training a model on separate mean abilities (*gM*) rather than on the average across competence domains (*gm*).

CBFAs enable the collection of data which can provide objective, quantitative and consistent estimates of students’ abilities. These assessments can be personalised and administered regularly, allowing to monitor individual progress without placing a significant workload on teachers. The use of ability estimates derived from rigorous model fitting of CBFA data that takes into consideration the difficulty of the items in an optimal manner, rather than the simpler scoring methods that are commonly used in educational settings for convenience, is a step forward towards improving the assessment of skill level. However, our models also showed bias, underestimating the ability of high achievers and overestimating the ability of low achievers (the opposite of what teachers have been reported to do, see (Demaray & Elliot, 1998). If the predicted abilities shrink towards the mean, this can have several significant implications in educational contexts. The most severe consequence arguably occurs when the model overpredicts low abilities, as this can result in students being given tasks that are too challenging or receiving less support than they need. Conversely, if high abilities are underpredicted, students may be misplaced and offered tasks that are below their capabilities. This can lead to a loss of motivation, incorrect perceptions of their abilities, and reduced future effort.

The systematic bias we observed can be reduced by increasing the amount of data available or by reducing the noise in the input data. If these options are not available, bias reduction can be achieved (at the cost of increasing the variance of the estimates), by, for example, rescaling the predictions using a separate training subset, or by making the distribution of the training data set more uniform, as we show in our simulations.

Although these systematic biases should not be ignored, a key advantage of predictions based on objective measures, such as those presented in this study, is that they may be less susceptible to irrelevant factors such as socioeconomic status, ethnicity or student personality traits, which have been shown to affect teachers’ appraisals (Alvidrez & Weinstein, 1999; Burgess & Greaves, 2013; Murphy & Wyness, 2020). They should also be more difficult to manipulate (either consciously or unconsciously) than grades (Diamond & Persson, 2016). Nevertheless, it is important to bear in mind that algorithmic predictions can still reflect biases present in the training data.

A broader question concerns the difficulty of predicting grades accurately. Given the crucial role in the educational system and in granting access to the job market, with significant long-term consequences for students’ future lives, it is troublesome that a large part of the variance in SA abilities could not be predicted even when combining FA and past SA scores. As some authors have pointed out (Anders et al., 2020), placing so much weight on a procedure whose determinants are largely unknown is problematic. This justifies further research on the predictors of educational attainment and the development of objective evaluation methods.

### Limitations

Some limitations of our study warrant mention. The data we analysed had been collected in advance, so we were unable to influence the design of the FAs to optimize the prediction. The large degree of sparsity of the sample decreases the reliability of the estimates, since extra variability is added by the fact that the estimates for different subject domains will be obtained from the responses to different item subsets. The selection of items, likewise, was not optimised for the purpose of prediction but reflected a mix of teacher choices and system recommendations, resulting, for instance, in the removal of a large amount of data because there were not enough features from different competence domains. Furthermore, the uncontrolled nature of the testing setting constitutes another source of uncertainty. Another caveat is that we only used simple dichotomous items that were suitable for automated scoring. More sophisticated item formats might be able to more accurately depict complex competences than those used here and enhance predictive power.

The estimation of the IRT parameters was performed for all students simultaneously (albeit separately for the FAs and the SAs), and therefore some information may be leaked between the train and the test data sets defined to evaluate performance. This problem is difficult to avoid, as the ability estimates of one student are based on the estimates of other students. Using only students in the test set to estimate their abilities would result in much worse estimates due to the much smaller data set size, and it would also have complicated the analysis pipeline and lengthened computation times substantially. The accuracy metrics that we present may be overestimating somewhat the actual model performance; notwithstanding, the impact of information leakage in this scenario should be minimal, and, as our main questions concerned the comparisons between models, our conclusions should be robust.

Finally, although our FA and SA data sets are both large, and the SA data set represents the entire population rather than a sample, we must acknowledge that they stem from a particular geographical region with a specific schooling system. The observed patterns may be influenced by the specific testing culture and educational context of the region under study. For instance, systems with a strong focus on high-stakes testing might show different FA-SA relationships due to greater washback effects, while systems with less emphasis on standardisation might demonstrate stronger correlations. A broader data set covering diverse educational systems would strengthen the validity and generalisability of our conclusions.

### Future Directions

Future efforts could attempt to introduce design choices (e.g., item selection) into FAs specifically with the purpose of enhancing predictive accuracy for student achievement, in addition to their usual monitoring role. Using data collected over longer periods or with higher frequency could provide more robust estimates of student development and potentially improve predictions, especially when previous summative assessment (SA) data are distant in time. Future research could also investigate whether more sophisticated item formats, capable of depicting complex competences, could enhance predictive power.

Another area for improvement will be to homogenise testing conditions to reduce uncertainty in the data, by controlling particular factors during testing sessions without increasing the burden on students, for example by conducting tests at regular times or ensuring that the environment meets specific requirements. An alternative option would be to record detailed information about the testing conditions (e.g., by asking examinees to report where a testing session has taken place) or other factors that may influence performance (e.g., demographic variables) which were not available here.

Lastly, while we propose strategies to mitigate the model biases we report, it will be important to investigate alternative solutions to this problem, given its potentially significant consequences.

## Conclusions

Owing to the important consequences of academic outcomes, it is critical to find objective measures of students’ abilities in ecological settings and with low burden to both students and teachers. Several advantages of FAs make them attractive candidates to support this endeavour, in particular their ability to collect intensive, longitudinal data with little interference on students’ daily activities. Our data show that, in spite of the multiple disparities between FAs and SAs, the former have substantial predictive validity for the latter. The ability of the best FA-based model to predict students’ abilities was comparable to published estimates of teachers’ ability to predict scores but still considerably lower than SA-based predictions. Considering separate specific abilities rather than a general ability was beneficial in terms of predictive value. Crucially, when basing decisions on CBFA models, the presence of biases and their trade-off with model accuracy should be borne in mind.

## Data Availability

This study’s data sets will not be made publicly available because Northwestern Switzerland’s four cantonal authorities (i.e., the contracting authorities) own them. Requests to access the data sets should be directed to the main office of the four cantons (kommunikation@bildungsraum-nw.ch).

## Footnotes

1 Grade 3 corresponds to the 5th year of mandatory schooling in the Swiss school system and to the age of 8 years.

## Code Availability

The code used for the analysis of the ability estimates and generation of the reported results is available on Github: https://github.com/benjamingarzon/checkspred. The analyses were not preregistered.
